# The Role of Macrophage Migration Inhibitory Factor in Alzheimer′s Disease: Conventionally Pathogenetic or Unconventionally Protective?

**DOI:** 10.3390/molecules25020291

**Published:** 2020-01-10

**Authors:** Maria Cristina Petralia, Giuseppe Battaglia, Valeria Bruno, Manuela Pennisi, Katia Mangano, Salvo Danilo Lombardo, Paolo Fagone, Eugenio Cavalli, Andrea Saraceno, Ferdinando Nicoletti, Maria Sofia Basile

**Affiliations:** 1 Department of Educational Sciences, University of Catania, 95124 Catania, Italy; m.cristinapetralia@gmail.com; 2 Department of Physiology and Pharmacology, University Sapienza, Piazzale A. Moro, 5, 00185 Roma, Italy; giuseppe.battaglia@uniroma1.it (G.B.); valeria.bruno@uniroma1.it (V.B.); 3 IRCCS Neuromed, Località Camerelle, 86077 Pozzilli, Italy; 4 Department of Biomedical and Biotechnological Sciences, University of Catania, Via S. Sofia 89, 95123 Catania, Italy; manuela.pennisi@unict.it (M.P.); kmangano@unict.it (K.M.); salvo.lombardo.sdl@gmail.com (S.D.L.); paolofagone@yahoo.it (P.F.); eugeniocavalli9@hotmail.it (E.C.); andreasara96@gmail.com (A.S.); sofiabasile@hotmail.it (M.S.B.)

**Keywords:** Alzheimer′s disease, macrophage migration inhibitory factor, neuroinflammation

## Abstract

Recent preclinical and clinical observations have offered relevant insights on the etiopathogenesis of late onset Alzheimer′s disease (AD) and upregulated immunoinflammatory events have been described as underlying mechanisms involved in the development of AD. Macrophage migration inhibitory factor (MIF) is a pleiotropic cytokine produced by several cells of the innate and adaptive immune system, as well as non-immune cells. In the present review, we highlight experimental, genetic, and clinical studies on MIF in rodent models of AD and AD patients, and we discuss emerging therapeutic opportunities for tailored modulation of the activity of MIF, that may potentially be applied to AD patients. Dismantling the exact role of MIF and its receptors in AD may offer novel diagnostic and therapeutic opportunities in AD.

## 1. Introduction

The term “dementia” refers to a cognitive decline that interferes with everyday life. The course of the disease is usually irreversible, and severely affects a patient′s social surroundings. Dementia represents an increasing public health, social, and financial problem due to population aging [1].

The most common form of dementia is accounted for by Alzheimer′s disease (AD) that includes between 50% and 75% of the cases of dementia with a doubling of its prevalence every five years after the age of 65 years [2,3]. AD is a neurodegenerative disease afflicting more than 45 million people worldwide with approximately 1% of those in their 60s and up to 8% of those above the age of 85. Although AD is prevalently sporadic, mutations in the three genes–amyloid precursor protein (APP), presenilin 1 (PSEN1), and presenilin 2 (PSEN2)–cause a rare (<0.5%) familial form of AD (fAD) that develops at an earlier age (between 30 and 50 years) than the sporadic case [2,3]. The remaining forms of the disease are due to vascular dementia (VaD), mixed Alzheimer′s and VaD, dementia with Lewy bodies, and frontotemporal dementia [2,3].

The progressive increase of the elderly population has been consequently accompanied by an increase in the prevalence of AD that is now favoring the adequate awareness of the relevance and the medical and social burden of AD [3].

Epidemiological and genetic studies, along with preclinical and clinical observations, have offered relevant insights able to dismantle the heterogenous nature of “typical” late onset AD. It is generally accepted that AD depends on a complex interaction of genetic and environmental factors, with the former taking account for ~70% of AD risk [3]. In particular, it is known that three variants, ε2, ε3, and ε4 of the APOE gene account for the highest risk of developing sporadic AD: in fact, ε4-carries have an odds ratio (OR) for AD of ~3 in heterozygotes and of ~12 in homozygotes, as compared to non-ε4 carriers [3]. Twenty additional genetic risk factors have been identified and are related to inflammation, cholesterol metabolism, and endosomal-vesicle recycling pathways [3]. The use of next generation sequencing has also revealed a number of other low frequency genes that confer relatively higher risk for AD, shedding light on a potential pathogenetic mechanism. Whilst each of these risk genes can, per se, confer a small increase in the OR, their combination in a polygenic risk score can almost double case prediction from chance [3].

**Figure 1:**
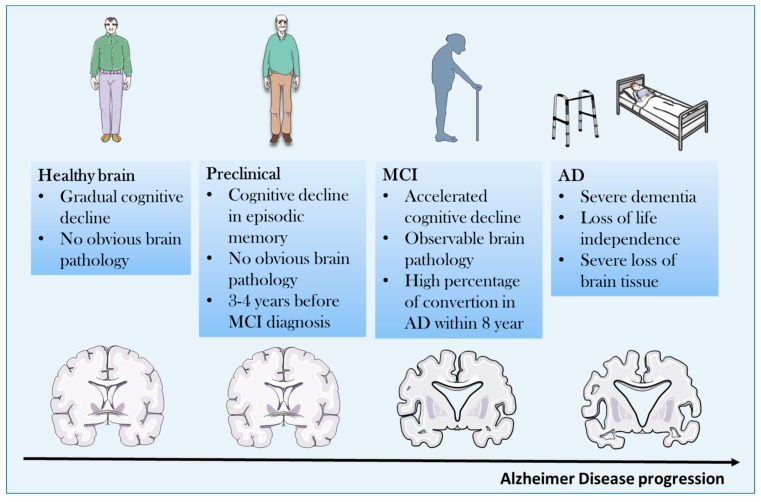
Clinical course of Alzheimer′s disease (AD) from asymptomatic to symptomatic stage. MCI: Mild Cognitive Impairment.

AD usually appears in a subacute manner in the preclinical phase characterized by reduced memory, cognition, and multiple personality changes. The course of the disease is usually progressive over a different period of time ranging from mild cognitive impairment to overt dementia that leads to complete cognitive impairment and physical disability and death, due to immobility [4,5] (Figure 1).

From the histopathological point of view, atrophy of the cerebral cortex and loss of neurons, associated with the presence of amyloid-β (Aβ) plaques and neurofibrillary tangles (NFTs), are the main features of AD [3]. Amyloid plaques are found in the extracellular space, and derive from a defective APP metabolism, as they are made of abnormally folded Aβ with 40 or 42 amino acids (Aβ40 and Aβ42) [3]. NFTs consist of hyperphosphorylated tau and they can be typically found in the entorhinal cortex and hippocampus and, only in later phases of the disease, in the associative isocortex [3]. Primary sensory-motor and visual areas are usually spared from NFTs. The severity of AD correlates with NFTs, while Aβ plaques reach a plateau early in the symptomatic phase of the disease [6].

In spite of several clinical studies conducted during the last 35 years, only five drugs have been approved by the FDA for treatment of AD which improve symptoms and slow down progression, but ultimately have little effect on the course and outcome of the disease. Four of the five FDA-approved AD drugs are acetylcholinesterase inhibitors: tacrine, donepezil, galantamine, and rivastigmine. However, because of its hepatotoxicity and the availability of better tolerated cholinesterase inhibitors, tacrine is now no longer used. The other approach for the treatment of AD is the inhibition of N-methyl-D-aspartate (NMDA) glutamate receptors, with consequent reduction of neuronal excitotoxicity. The only NMDA receptor inhibitor approved for use in AD is memantine [1,7,8].

## 2. Pathogenetic Concepts

There are at least four major pathogenetic players in the development of AD [2]. Given the heterogeneity of the disease, each of them may contribute differently to the induction, onset, and progression of the disease in individual cases. These hypotheses include the amyloid cascade hypothesis, the tau hypothesis, the cholinergic hypothesis, and the excitotoxicity hypothesis. Although each of them has also been evaluated as a potential therapeutic target, the clinical outcomes have been disappointing [1] for several possible reasons. For example, intriguing proposals of the reasons beyond the failure of specific targeting of the amyloid hypothesis and the tau hypothesis in the clinical setting are elegantly presented in a recent review [8].

There are also other well promoting factors in the pathogenesis of AD including hyperinsulinemia and diabetes, as well as the occurrence of accompanying neuroinflammation [2]. It has also recently been proposed that a common pathogenetic pathway connecting diabetes, subchronic inflammation, and AD may rely on the activation of the common pathogenetic pathway consisting of PTN-MK-RPTPβ/ζ Axis [9]. Strengthening the causal link between deregulated glycaemic homeostasis, including insulin secretion and resistance and AD, is the recent identification of a newly indicated form of diabetes named “type 3” diabetes that is characterized as a brain-insulin resistant state linked to AD [10]. Although the precise cellular type that develops insulin resistance in the brain has not yet been identified, recent studies suggest that cortical neurons develop resistance to insulin after prolonged exposure to high insulin concentrations. This occurs through inhibition of phosphorylation of Akt, p70S6K, and GSK-3β [11]. As MIF inhibits Akt phosphorylation its high expressions during brain pathologies may exacerbate insulin resistance in neurons [12].

We have previously discussed that the Apoε4 (Apoε4) allele of the apolipoprotein (apo) gene, has also been identified as the primary genetic risk factor for AD [1,2].

## 3. Neuroinflammation and AD

That upregulated immunoinflammatory events may play an important pathogenetic role in the development of AD is supported by several preclinical and clinical observations and this area of research has gained much interest during the last decade.

### 3.1. Innate Immune System and AD

The better understanding of the functioning of the innate immune system, with the emerging role played from interaction of danger associated molecular pathway (DAMP) with pathogen recognition receptors (PRR) on cells of the innate immune system has allowed us to gain important insight into immune pathogenetic concepts of AD. It is proposed that during the development of AD misfolded and aggregated proteins might act as DAMP that binds pathogen PRR including class A scavenger receptor A1, CD36, CD14, α6β1 integrin, CD47, and toll like receptors expressed by microglial cells and astrocytes. This would in turn determine the release of inflammatory mediators such as NO and ROS, as well as proinflammatory cytokines, including TNF-α and IL-1β [13,14,15,16].

### 3.2. Proinflammaory Cytokines and AD

It has therefore been proposed that pro-inflammatory cytokines may contribute to AD pathogenesis through multiple pathways, including the induction of indoleamine 2,3-dioxygenase that increases the levels of the quinolinic acid, a neurotoxic factor, and in turn promotes tau hyperphosphorylation [13,14,15,16]. The role of anti-inflammatory cytokines, primarily belonging to the Th2 and Th3 cell subsets in the process of AD, is also worth mentioning as they might exert protective effects against AD by counteracting the effects of pro-inflammatory cytokines [13,14,15,16]. For example, transforming growth factor (TGF)-β that is a prototypical anti-inflammatory cytokine produced by Th3 cells is capable of ameliorating Aβ-induced cytotoxicity, in vivo and in vitro, and deficiency of TGF-β1 promotes both Aβ accumulation and NFTs formation [13,14,15,16]. This pathogenetic hypothesis is also consistent with the epidemiologic evidence indicating an affirmative influence of non-steroidal anti-inflammatory drugs (NSAIDs) on delaying the progression of AD and have suggested that a blockade of the ongoing inflammatory processes may delay the progression AD [1].

It is important to note that despite local recruitment of brain microglia to the sites of amyloid deposition, they ultimately fail in preventing the formation of β-amyloid plaques [13,14,15,16].

Several studies have reported that pro-inflammatory cytokines are augmented in patients with AD, and genetic polymorphisms for these cytokines have also been reported but often remain unconfirmed in patients with the same or different ethnicity [17].

Accordingly, though with some contradictory findings, prototypical pro-inflammatory cytokines of the innate immune system including IL-1β, TNF-α, IL-6, IL-12, and IL-23 have been found augmented in plaques and or CSF of AD patients and animal models of the disease. In transgenic mouse models of AD, inflammatory cytokines correlate with amyloid load [17].

In addition, an antibody directed against IL-12/IL23 ameliorates the course of the disease in a mouse model of AD [18].

### 3.3. Proinflammatory Cytokines as Biomarkers During AD Development and Progression

Recent studies have also shown that immune pro-inflammatory cytokines, such as macrophage migration inhibitory factor (MIF) and YKL-40, TNF receptors and sTREM2, are associated with tau pathology and brain aging, thus suggesting that these molecules may be useful diagnostic markers and therapeutic targets [19].

Another study showed that the CSF markers -IL-15, MCP-1, VEGFR-1, sICAM1, sVCAM-1, and VEGF-D- are independently associated with the CSF tau and p-tau181 levels [20]. Together with the observation of Bacher and colleagues [21] that MIF is augmented in AD, these data may suggest that measuring blood levels of MIF may represent a diagnostic biomarker that may be useful both for diagnosis and therapeutic monitoring of the disease, at least in a well-defined subset of patients that are characterized by larger production of MIF, and that may also be considered for tailored therapeutic approaches with specific MIF inhibitors. However, longitudinal or perspective studies on the possible use of MIF as diagnostic biomarkers are so far missing.

The pathogenetic relevance of neuroinflammation in AD models is highlighted from a systematic review that reports beneficial effects on the course of experimental rodent models of AD with several immunomodulatory agents including IL-1 receptor antagonist and TNF inhibitors [22].

As an important note to the neuroinflammatory hypothesis of AD, it is worth noting that the inability of plaque clearance by CNS phagocytes, along with upregulated inflammation, seems instrumental to disease progression. Accordingly, several genes associated with sporadic AD are involved in glial clearance of misfolded proteins. Also, external factors, i.e., systemic inflammation and obesity, promote disease development and evolution [23,24].

Simultaneously, and along this line of research, it is interesting to observe that peripheral phagocytes are able to effectively clear plaques and therapeutic strategies aiming at favoring recruitment of these cells into the CNS are actively being pursued. It has been suggested that Aβ immunotherapy could clear cerebral Aβ accumulations by activating phagocytes, and recent evidences generated in preclinical AD models propose that targeting the TGF-β-Smad 2/3 signaling is able to modulate blood-to-brain trafficking of these cells [25,26]. Also, the chemokine receptor Cx3cr1 pathway seems to control the chemotaxis of phagocytes toward AD affected neurons [25,26].

## 4. MIF: An Emerging Player in the Neuroinflammatory Hypothesis of AD

### 4.1. Biology, Physiology and Physiopathology of MIF

MIF is a pleiotropic cytokine produced by several cells of the innate and adaptive immune system, as well as non-immune cells including myocardial cells. MIF has been discovered at the end of the 60 and its name is due to its ability to inhibit the migration of macrophages. MIF has also attracted much attention because of its unique interaction with corticosteroids [27].

MIF has been shown to overcome the inhibitory effects of glucocorticoids on the production of pro-inflammatory cytokines by monocytes in vitro and to counteract steroid effects against lethal endotoxemia in vivo. MIF also counteracts glucocorticoid inhibition of T-cell in vitro by restoring the expression of IL-2 and interferon (IFN)-γ and may therefore play a major role in determining resistance to steroids. Through its ability to interact with steroids, MIF is also endowed with hormone-like properties and it has been shown to modulate the HPA axis [28].

Recent studies have highlighted the mode of action and signaling transmission of MIF, as well as its complex role played in regulation of immune responses.

MIF transduces its biological signals by binding to the CD74 receptor or the co-receptors, CXCR2, CXCR4, and CXCR7. In turn, a variety of signaling cascades, including the MAPK, PI3K/AKT, and NF-kB pathways are activated. By doing so, MIF activates pro-inflammatory events including the secretion of IL-6 and TNF-α and the activation of the inflammasome [29,30,31].

For this reason, MIF has been implicated in the pathogenesis of several autoimmune diseases including type 1 diabetes, multiple sclerosis, autoimmune hepatitis, and rheumatoid arthritis [32,33,34,35,36].

MIF may also contribute to immunoinflammatory events during development of Duchenne′s syndrome [37] and seems to play a pathogenetic role in major depressive disorders [38]. More recent studies also indicate that MIF is implicated in certain forms of cancer phenotypes such as melanoma, glioblastoma, prostate cancers, and neuroblastoma [39,40,41,42,43,44,45].

However, and in a manner similar to other cytokines with a primarily pro-inflammatory profile, MIF might also display anti-inflammatory activities that appear to be primarily mediated by its ability to activate AMPK and inhibit the JNK pathway. These effects may be taken into account by the capacity of MIF to reduce the development of an immunoinflammatory condition such as cardiac ischemia/reperfusion [46,47].

### 4.2. The Emerging Role of MIF Homologue, D-dopachrome Tautomerase (D-DT; MIF-2)

Adding interest to the role of MIF, a second structurally related MIF family member, D-DT, was recently characterized [48]. D-DT or MIF-2 was recognized to be a structural and functional homolog of MIF, which could exert overlapping effects, further raising the complexity of canonical MIF signaling pathways [48].

D-DT often, but not always, exerts synergistic and overlapping effects of MIF. Both MIF and D-DT play a key role in progressive forms of MS in male patients and D-DT has also been found to exert oncogenic effects [33,39].

## 5. MIF in Neurodegenerative Diseases

### MIF in PD and ALS

The role of MIF in neurodegenerative diseases has also received much attention (Table 1). In agreement with its pleiotropic biological functions, emerging evidence indicates that MIF may play a complex role in neurodegenerative disorders with potential beneficial effects in Parkinson′s Diseases (PD) and amyotrophic lateral sclerosis (ALS).

In particular, we have first shown that PD patients have elevated blood levels of MIF that do not correlate with severity of the disease [49]. On the basis of some beneficial effects observed with exogenously administered MIF in models of PD, we postulated that this increase might have reflected a compensatory attempt to counteract pathogenetic pathways in the course of the disease, and that endogenous MIF might have played a beneficial role in PD [49].

Recent preclinical studies have confirmed that endogenous MIF plays protective anti-inflammatory roles in a mouse model of PD via inhibition of apoptosis and induction of autophagy [50].

A protective role for endogenous MIF has also been proposed for ALS since the original in vitro observation that MIF inhibited mutant SOD1 misfolding and that MIF in mutant SOD1-expressing motor neurons downregulated the accumulation of misfolded SOD1 increasing cell survival [51]. Subsequent studies from the same group have shown that the inhibitory effects of MIF on misfolded SOD1 were independent of its cytokine activity and due to its cytosolic chaperone-like properties [52].

Studies from the same group have validated the potential beneficial effects of endogenous MIF in ALS by demonstrating that knockdown of MIF gene was associated with higher amounts of misfolded SOD1 in the spinal cord, and consequently accelerated disease onset and shortened the lifespan of mutant SOD1 mice [53,54]. Conversely, when overexpression of MIF was achieved in the spinal cords of mutant SOD1^G93A^ and loxSOD1^G37R^ mice by adeno-associated viral (AAV) vectors a significant delay in disease onset and prolonged survival [54].

Taken as a whole, these data prove that MIF has a pivotal role in the folding of SOD1, and they support the potential therapeutic role of up-regulating MIF within the CNS to modulate the selective accumulation of misfolded SOD1.

## 6. MIF and AD

In spite of these beneficial neuroprotective effects of MIF in PD and ALS, conflicting data have been generated as to whether MIF plays a harmful or beneficial role in AD. Until a few years ago, there was general agreement that MIF plays a key role as pathogenetic cytokine in AD pathogenesis and progression. Two recent papers have, however, questioned this view and propose that endogenous MIF may be beneficial in AD and that its augmented levels found in post mortem brains, CSF, and periphery of AD patients witness a compensatory attempt at counteracting insufficient biological function of MIF in the brain due to glycation, oxidation, and binding to plaques.

We will present here findings of experimental, human genetic, and clinical studies that have studied MIF in rodent models of AD and AD patients (Table 2), and will highlight emerging therapeutic opportunities for tailored modulation of the activity of MIF that may potentially be applied to AD patients. Dismantling the exact role of MIF and its receptors in AD may offer novel diagnostic and therapeutic opportunities in AD This focused review may also propel further interest on additional studies of the yet unexplored role of D-DT in AD.

### 6.1. Preclinical Studies

In-silico generated data suggested that MIF may be implicated in the regulation of neuronal apoptosis during AD [55]. Also, in-vitro dose-dependent dichotomic effects for MIF were observed, as low concentrations of increased the ratio of p-Bad/Bad, while high levels of MIF induced the expression of Bad and triggered cell apoptosis [55]. Interestingly, MIF exhibited a pattern of neurotoxicity similar to Aβ_1–42_, which was associated with the MIF-induced increased expression of Bad [55].

In another in-vitro study it was shown that the small molecule inhibitor of MIF ISO-1 protected PC12 cells from advanced glycation end product (AGE) aggravation of PC12 cell injury induced by Aβ_1–40_ [56]. This finding may indicate that AGE may represent a pathogenetic link between type 2 diabetes and AD, and that MIF may be a common pathogenetic mediator [56].

By using the APP transgenic mouse model, Bacher et al. observed that serial brain sections of transgenic APP mice expressed MIF immunolabeling in microglial cells in association with Aβ plaques [21]. In addition, in-vitro studies in murine and human neuronal cell lines showed that Aβ-induced toxicity was significantly reverted by a small molecule inhibitor of MIF (ISO-1) [21].

However, another study conducted in transgenic Tg2576 mice expressing high levels of the Swedish double mutation of human amyloid precursor protein that develops typical β-amyloid plaques in the brain cortex failed to detect MIF mRNA in several cortical areas [57]. It should, however, be noted that in this model only IL-1β was found to be induced in reactive astrocytes surrounding β-amyloid deposits detected in 14-month-old Tg2576 mice out of several cytokines tested, including IL-1α, IL-6, IL-10, IL-12, IL-18, IFN-γ, TNF-α, and macrophage chemotactic protein (MCP)-1 [57]. On the basis of these findings, the authors suggest that the local immune response detected around cortical β-amyloid deposits in transgenic Tg2576 mouse brain may be different from that observed in brains from AD patients [57].

The pathogenetic potential of MIF predicted from these in-vitro studies were confirmed in a study conducted in MIF knockout (KO) APP/PS1 transgenic mice [58]. By using immunofluorescence staining the authors found that MIF deficiency attenuated tau hyperphosphorylation in mice receiving intracerebroventricular injection of streptozotocin as compared to wild-type (WT) mice [58]. In addition, the authors cultured primary astrocytes from both MIF KO and WT mice in the presence of high glucose to mimic STZ function in vitro. They then treated neurons in vitro with either of the condition mediums (CM) [58]. CM from high glucose-treated WT astrocytes increased tau hyperphosphorylation in the cultured primary neuron and the effect was prevented by the MIF inhibitor ISO-1 [58]. In a similar manner, hyperphosphorylation was not observed in cultured primary neurons treated with CM from *Mif^−/−^* astrocytes [58].

### 6.2. Clinical Studies

#### 6.2.1. Genetic Polymorphism

Two polymorphisms in the promoter region of MIF-rs755622 and rs5844572-exhibit prognostic relevance in inflammatory diseases. In two Italian studies, variations of MIF-173 G > C (rs755622) were not associated with AD [59,60].

#### 6.2.2. Circulating Levels of MIF in Blood and CSF

A study by Bacher et al. showed significantly higher MIF levels in the CSF of AD patients and mild cognitive impairment (MCI) subjects [21]. Also, Oyama et al. reported that MIF colocalized with Aβ in the brains of patients, and therefore the toxicity of Aβ was seemingly to be attributed to the upregulation of MIF expression [61].

Another study conducted from a Korean group also demonstrated that plasma levels of MIF were augmented in patients with both MCI and AD in comparison to healthy controls [62].

Finally, a study from a German group confirmed in 2009 that elevated CSF levels of MIF were observed both in patients with MCI and AD as compared to subjects without cognitive deficits [63]. The authors observed that in the AD group the levels of MIF did not differ between the patients with mild dementia (defined as MMSE score >20) and the patients with moderate or severe dementia [63].

Taken as a whole, these data indicate increased MIF production in AD and MCI, suggesting that MIF may be involved in the occurring neuroinflammatory process at a clinical pre-dementia disease stage. The data, however, indicate that there is no direct correlation between MIF levels and the severity of the disease.

## 7. Contrarian Thinking: Augmented levels of MIF Are Secondary to Local Insufficient Biological Activities of MIF and Represent a Homeostatic Attempt to Revert AD Progression

### 7.1. Oxidized and Reduced Isoforms of MIF

Recent studies have shown that circulating MIF can occur in two forms: oxidized MIF (oxMIF) and reduced MIF (redMIF) [64,65]. Accordingly, RedMIF is the isoform that can be more abundantly expressed, and is also detectable in healthy subjects, whereas oxMIF represents the disease-related isoform which is predominant in the blood and on the surface of cells isolated from patients suffering from chronic inflammatory disorders and cancer [64,65].

It is of interest that the monoclonal anti-oxMIF antibodies BaxB01, BaxG03, and BaxM159 can differentiate between redMIF and oxMIF, and exert protective effects in animal models of inflammation [64,66].

### 7.2. Dysregulayed Balance of Oxidized and Reduced Isoforms of MIF in AD

Along this line of research using an early glycation profile of human brain by fluorescent phenylboronate gel electrophoresis Kassaar et al. identified early glycation and oxidation of MIF in the AD brain [67]. This modification inhibits MIF enzyme activity and ability to stimulate glial cells. On the basis of this finding, they hypothesize that MIF in the AD brain can be both glycated and oxidized. The authors propose that the demonstrated inability of glycated and oxidized MIF in stimulating glial cells in vitro may represent an important primum movens in defective clearance of plaques from CNS phagocytes during the development of AD. Inferring from this, the augmented CSF and peripheral levels may be part of a homeostatic attempt that, in an unsuccessful way, ultimately aims at counteracting the endogenous functional deficiency of cerebral glycated and oxidized MIF [67].

Lending support to the concept that MIF may be protective in AD, a recent study has found that MIF expression was upregulated in the brain of AD patients and animal models [68]. In agreement with previous studies discussed above, MIF was detected in the CSF of AD patients, but not in that of the patients with MCI and vascular dementia [68]. It should, however, be noticed that neither does this study discriminate between RedMIF and oxMIF. The authors also studied expression and function of MIF in the transgenic model of AD that can be observed in APP23/PS45 double transgenic mice that develop a significant amount of plaques and cognitive impairments [68]. To evaluate the effects of reduction of endogenous MIF during the course of the disease, these mice were bred with *Mif*^+/−^ mice on C57/BL6 background. The authors demonstrated that in a manner similar to AD patients, MIF was also upregulated in the brains of these double transgenic mice at 3 months of age when a large amount of amyloid plaques had been formed [68]. MIF expression largely associates with Aβ deposits and microglia [68]. The authors further studied the role of endogenous MIF in APP23/MIF^+/−^ and APP23 mice in cognitive performance during AD pathogenesis [68]. Although hemizygous knockout of MIF did not affect mouse mobility or vision, APP23/MIF^+/−^ mice showed significant memory impairment, indicating that MIF deficiency affected spatial learning [68]. Further, it was found that the Aβ treatment of SH-SY5Y cells induced a significant increase of MIF concentrations in the culture medium thus suggesting that Aβ-triggered MIF secretion by neurons [68]. Finally, it was also found that Aβ-induced toxicity that dramatically reduced the cell survival rate in SH-SY5Y cells was completely prevented in the SH-SY5Y cell line stably overexpressing MIF, suggesting that elevated MIF secretion protects neurons from Aβ-induced cytotoxicity [68].

In light of these findings and in agreement with previous data on the neuroprotective action of MIF in models of cerebral ischemia/reperfusion associated with reduced caspase-3 activation [69], the authors suggest that at the late stage of AD, a large portion of the extracellular MIF is sequestered by Aβ plaque, which impedes its cerebral biological functioning and is followed by an unsuccessfully compensatory attempt [69]. This compensatory attempt would ultimately activate a vicious circuit of immunoinflammation both within the CNS and in the periphery that further aggravates the progression of AD pathology.

## 8. Conclusions

**Figure 2:**
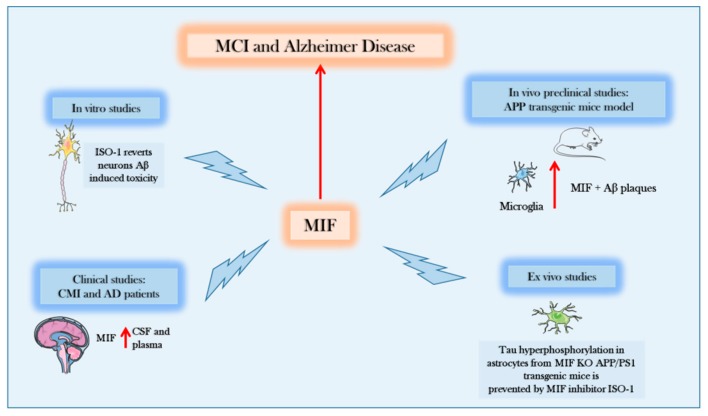
Evidences supporting the role of Macrophage Migration Inhibitory Factor (MIF) in Alzheimer′s disease (AD). In-vitro, in-vivo, and ex-vivo preclinical data, as well as data from AD and MCI patients show that MIF is increased during the course of the disease and that therapeutic intervention aimed at counteracting MIF function may exert beneficial effects on AD course. MCI: Mild Cognitive Impairment; Ab: Amyloid Beta; APP: Amyloid Precursor Protein; PS1: Presenilin 1; CSF: CerebroSpinal fluid; KO: Knockout; ISO-1: 4,5-Dihydro-3-(4-hydroxyphenyl)-5-isoxazoleacetic acid methyl ester.

The present review highlights the involvement of endogenous MIF in the pathogenesis of AD. Most of the findings generated until 2017 favored the hypothesis that this cytokine played a key role in the initiation and progression of the disease (Figure 2).

These findings were corroborated by in-vitro and in-vivo findings demonstrating protection from tau phosphorylation in MIF KO transgenic models of AD [58] and elevated levels of MIF in periphery and CSF of both AD patients with MCI and more severe diseases [62,63]. However, as indicated, a correlation between CSF levels of MIF and disease severity was not found [63]. The observation that MIF levels also correlated with tau and aging lent further support to the proposed pathogenetic role of MIF in AD.

As discussed above, along with other cytokines and immunoinflammatory mediators MIF reproducibly associated with tau and aging, thus suggesting that it could represent a useful diagnostic marker and therapeutic target [19].

The recent observations that the glycated and oxidized MIF found in the brain of AD patients [67] is unable to exert its activation function in glial cells, thus favoring plaque clearance, has suggested a new view on the role of MIF in AD, and more specifically that the augmented levels could be a compensatory attempt aimed at overcoming insufficient biological function. The more recent study performed by Zhang and coworkers seems to support this view even if in an independent manner from biological deficiency of MIF secondary to glycation and oxidation. They would rather propose that MIF is absorbed in the Aβ plaques, preventing its protective functions on neurons [68].

There are clear conflicting results between the two opposite hypotheses that are difficult to reconcile, including the beneficial effect of MIF deficiency on tau phosphorylation in one AD transgenic model with the protective effect of endogenous MIF in another transgenic model.

In addition, there is a clear contrast between in-vitro findings from Bacher et al. who demonstrated that the MIF inhibitor ISO-1 reverted Aβ-induced toxicity in SH-SY5Y cells [21] with that from Zhang et al. that Aβ-induced toxicity was completely prevented in SH-SY5Y cell line stably overexpressing MIF (SYMS) [68].

Future studies are needed to dismantle the role of endogenous MIF in AD to entail the use of specific MIF inhibitors and exogenously administered MIF in transgenic models of AD, as well as the dosing of RedMIF vs. oxMIF in CSF and periphery of AD patients at different stages of the disease. Studies are also strongly warranted on the potential role of the second member of the MIF family, D-DT on the development of AD. Studies of D-DT in either rodent models or AD patients are lacking so far.

Understanding whether MIF plays a pathogenetic or beneficial role in AD is important for tailored therapeutic approaches. In the former case, tailored inhibitors of MIF that are already in the clinical setting could be considered for pilot studies, which include the tautomerase inhibitor ibudilast that is marketed for different indications and is being repurposed for immunoinflammatory diseases [70], as well as anti-MIF mAb that have completed Phase I/II testing in cancer patients and the anti-CD74 mAb milatuzumab that is approved for patients with multiple myeloma [71]. It is also worth noting that the biological function of MIF can be inhibited by nitrosylation [72], and hence nitric oxide (NO) donors may indirectly promote MIF inhibition. Along these lines, we propose that together with NO-NSAID, that have been proven effective in both preclinical and clinical settings [73,74], and also other NO-donors such as the recently characterized lopinavir-NO and ritonavir-NO [75,76,77,78], deserve consideration as drugs to be used in the treatment of AD patients. These drugs NO donors could combine beneficial anti-inflammatory properties along with beneficial effects of NO on vascular conditions associated with AD. It is indeed noticeable that lopinavir-NO has been shown to be able to successfully prevent a model of MIF-dependent immunoinflammatory hepatitis [79,80].

Vice versa, if endogenous MIF plays a protective role in the development of AD, efforts should be made to tailor agonistic approaches consisting of either MIF itself or active peptides in the early phase of the disease. In a similar manner, ad hoc safety studies should be carried out in patients that are treated with specific MIF inhibitors for different indications to prevent possible contribution to the activation of AD, especially in individuals with a high risk phenotype. In this regard, preclinical studies in rodent models should be carried out to establish whether specific MIF inhibitors jeopardize cognitive functions [58].

## Figures and Tables

**Table 1:** Evidences for the involvement of the Macrophage Migration Inhibitory Factor (MIF) in neurodegenerative disorders.

Disease	Preclinical Data	Human Data	References
Parkinson′s Diseases (PD)	MIF reduces apoptosis and induces autophagy in an in vitro model of PD (SH-SY5Y cells exposed to MPP+)		[50]
	MIF is upregulated in mouse model of PD (induced by i.p. injection of MPTP)		[50]
		↑ serum levels	[49]
Amyotrophic Lateral Sclerosis (ALS)	MIF inhibits mutant SOD1 misfolding in motor neuron-like cells		[51,52]
	Endogenous MIF knockdown in SOD1 mutant mice accelerates disease		[53,54]
	MIF overexpression in the spinal cord improves ALS in SOD1 mutant mice		[53,54]

MPP+: 1-methyl-4-phenylpyridinium; MPTP: 1-Methyl-4-phenyl-1,2,3,6-tetrahydropyridin; i.p.: Intra-peritoneal; SOD1: superoxide Dismutase 1.

**Table 2:** Evidences for the involvement of the Macrophage Migration Inhibitory Factor (MIF) in Alzheimer′s Disease (AD).

Preclinical Data	Human Data	References
ISO-1 reduces Aβ-induced toxicity in vitro		[21,61]
	↑ MIF in CNS of AD and MCI patients	[21,61]
MIF colocalizes with Aβ plaques in APP23 transgenic mice		[21,61]
	↑ MIF in plasma of AD and MCI patients	[62]
MIF deficiency attenuates tau hyperphosphorylation in astrocytes from APP/PS1 transgenic mice		[58]
MIF overexpression prevents Aβ toxicity in SH-SY5Y cells		[68]
APP23/MIF^+/−^ mice show significant memory impairment		[68]
